# Voluntary peak cough flow: A simple and effective tool to predict dysphagia across diverse etiologies

**DOI:** 10.1007/s00405-026-10037-x

**Published:** 2026-02-16

**Authors:** Hakan Gölaç, Güzide Atalık, Adnan Gülaçtı, Ebru Şansal, Banu Tijen Ceylan, Metin Yılmaz

**Affiliations:** 1 Department of Speech and Language Therapy, Faculty of Health Sciences, Gazi University, Ankara, Türkiye; 2 Department of Otolaryngology, Faculty of Medicine, Gazi University, Ankara, Türkiye

**Keywords:** Cough, Deglutition disorders, Dysphagia, Quantitative measurements

## Abstract

**Background and aim:**

Swallowing and coughing share neural and muscular substrates and play crucial roles in airway protection. This study aimed to compare voluntary peak cough flow (PCF) between patients with various dysphagia etiologies and healthy controls, and to identify a PCF cutoff value for predicting dysphagia.

**Methods:**

A total of 90 participants were included in the present case-control study. The study group (SG) consisted of 45 patients with FEES-confirmed dysphagia (M/F: 25/20) with a mean ± SD age of 66.64 ± 12.97, and the control group (CG) consisted of 45 age- and gender-matched healthy volunteers (M/F: 25/20) with a mean ± SD age of 65.56 ± 16.39. All participants underwent voluntary PCF measurement using an analog peak flow meter. The cough trials were performed five times for each subject, and the highest value obtained across the trials was recorded as PCF.

**Results:**

The SG exhibited significantly lower PCF compared with the CG (206.11 ± 74.22 vs. 287.78 ± 89.31 L/min, *p* < 0.001). Receiver operating characteristic (ROC) analysis identified a PCF cutoff value of ≤ 252.5 L/min for distinguishing dysphagia, with an area under the curve (AUC) of 0.765 (95% CI: 0.666–0.865), sensitivity of 71.1%, and specificity of 71.1%.

**Conclusions:**

Voluntary PCF was significantly reduced in individuals with diverse dysphagia etiologies compared to healthy controls and showed moderate discriminant ability to predict dysphagia. Given its simplicity, low cost, and portability, PCF measurement may serve as a practical adjunctive tool in predicting dysphagia, particularly where instrumental swallowing evaluations are not feasible.

## Introduction

Swallowing and coughing are vital physiological functions that contribute to airway protection and help maintain airway integrity by preventing and clearing penetration/aspiration [1]. While swallowing facilitates the safe transport of food and liquid into the esophagus, an effective cough mechanism acts as a primary line of defense against the airway invasion of foreign materials. The intricate coordination between these two functions is governed by shared neural pathways and muscular structures, particularly in the brainstem and upper aerodigestive tract [2, 3]. Impairments in swallowing function (dysphagia) can disrupt adequate airway protection, increasing the risk of airway invasion. Similarly, disordered cough function (dystussia) may compromise the clearance of penetrated/aspirated material into the airway, further exacerbating the consequences of dysphagia. Accordingly, a parallel deterioration in both functions is commonly observed in individuals with dysphagia [4–6].

Objective dysphagia assessment encompasses the use of standardized, measurable, and instrument-based methods to evaluate swallowing physiology and airway safety, providing quantifiable data that enhance diagnostic accuracy and reduce subjectivity [7, 8]. Videofluoroscopic swallow study (VFSS) and fiberoptic endoscopic evaluation of swallowing (FEES) are widely regarded as the gold-standard tools for the instrumental assessment of swallowing disorders. However, the limited availability of resources, challenges with patient compliance, and the need for specialized expertise restrict the routine use of these instrumental examinations for patients with dysphagia in daily clinical practice [9–11]. Therefore, given the close relationship between swallowing and cough functions, using cough-related measurements as a quantitative tool during clinical swallowing assessments may offer a valuable dimension for evaluating airway protection and respiratory-swallow coordination [12].

Coughing may be either voluntary, initiated by the cerebral cortex, or reflexive, triggered by activation of airway sensory nerve receptors [13]. In fact, an increasing body of literature highlights the coexistence of swallowing and cough impairments, with growing attention to the predictive value of cough for swallowing dysfunction. While the majority of those studies were conducted with patients with neurogenic dysphagia, including stroke [6, 14–17], Parkinson’s Disease (PD) [5, 18–23], and amyotrophic lateral sclerosis (ALS) [4, 24, 25], some others included patients with head and neck cancer [26, 27] and even those with cervical spinal cord injury [28]. It is important to present specific cough characteristics in homogeneous patient populations, but it is also essential to provide the results of cough measurements in patients with various dysphagia etiologies. However, only a few studies have examined the link between voluntary cough performance and objective swallowing outcomes in a heterogeneous dysphagia population [29, 30].

The measurement of peak cough flow (PCF) is one of the most robust methods for objectively measuring voluntary cough efficiency. Measuring PCF with a digital or analog peak flow meter has been used in numerous studies as a cough assessment tool for patients with dysphagia, because of its affordable, easy-to-use, portable, and commercially available nature [15, 17, 19–22, 24, 27, 29, 30]. Given the wide range of dysphagia etiologies encountered in clinical practice, incorporating user-friendly and straightforward cough measurements into routine assessments may improve the sensitivity of evaluations and enable more accurate identification of patients with dysphagia. In light of the prior studies, we hypothesized that patients with confirmed dysphagia with a wide range of etiology would have reduced voluntary PCF than healthy individuals. Therefore, the present study aimed (1) to compare the PCF between patients with confirmed dysphagia and their age- and gender-matched healthy volunteers using a low-tech peak flow meter device and (2) to provide a cutoff PCF value for patients with diverse dysphagia etiologies.

## Materials and methods

### Participants

This case-control study was approved by the research ethics committee of Gazi University (IRB number: 903) and conducted in accordance with the Declaration of Helsinki. In total, 126 consecutive patients with suspected dysphagia who were referred to our dysphagia clinic for a detailed swallowing examination were assessed using FEES. Among them, 81 patients were excluded based on the the following exculision criteria (1) age under 18 years; (2) having a tracheostomy cannula at the time of assessment; (3) unable to follow instructions because of impaired conscious state or severe cognitive dysfunction; (4) smoking within the previous 5 years; (5) having a recent respiratory event or a history of any disease that could affect respiratory system (e.g., chronic obstructive pulmonary disease, asthma, lung cancer); (6) not having a deteriotation in swallowing efficiency or safety based on FEES findings; (7) those with vocal fold paralysis, which may result in glottal insufficiency. A total of 45 patients with FEES-confirmed dysphagia (M/F: 25/20) were included as the study group (SG). In addition, 45 age- and sex-matched healthy volunteers (M/F: 25/20) were recruited to form the control group (CG). Inclusion criteria for the CG were: (1) age ≥ 18 years; (2) absence of any current or previous complaints of swallowing difficulties; (3) no history of neurological, structural, or respiratory conditions that could influence swallowing or cough performance. Individuals were excluded if they were active smokers within the past 5 years or exhibited any clinical signs suggestive of impaired swallowing efficiency or safety during a preliminary screening. All participants were informed about the study, and written informed consent was obtained from each before enrollment.

### Swallowing study

The dysphagia was confirmed based on a comprehensive FEES in the SG. During FEES, participants were seated in an upright comfortable position. An Olympus ENF-GP flexible laryngoscope (Tokyo, Japan) and Atmos Cam 21 endovision camera system (Lenzkirch, Germany) were used with no topical anesthetic or vasoconstrictor to avoid disrupting the natural swallowing function. Anatomical and functional features of the oropharyngeal and laryngeal structures were assessed qualitatively during FEES; however, they were not systematically documented for subsequent analysis. Bolus consistencies were identified based on the International Dysphagia Diet Standardisation Initiative (IDDSI) framework [31]. The swallowing trials consisted of thin liquid (IDDSI level 0) and mildly thick liquid (IDDSI level 2) presented in volumes of 5 mL, 10 mL, and cup sip; extremely thick liquid (IDDSI level 4) administered as one tablespoon; and regular food (IDDSI level 7) provided as a standard-sized cracker. The Yale Pharyngeal Residue Severity Rating Scale (YPR-SRS) [32, 33] and the Rosenbek’s Penetration–Aspiration Scale (PAS) [34] were used to evaluate swallowing efficiency and swallowing safety, respectively. The number of swallowing trials varied across participants depending on their swallowing performance during FEES. For example, if laryngeal aspiration was observed after a 5 mL of mildly thick liquid (IDDSI level 2), no trials were provided with lower (i.e., IDDSI level 0) consistency. The worst YPR-SRS and PAS scores out of all bolus trials for each consistency were noted. After a careful FEES examination by an ENT specialist and a speech and language therapist (SLT), dysphagia was confirmed if the patients had a YPR-SRS and/or PAS score of ≥ 3 in any of the tested consistencies.

### PCF measurement

**Fig. 1:**
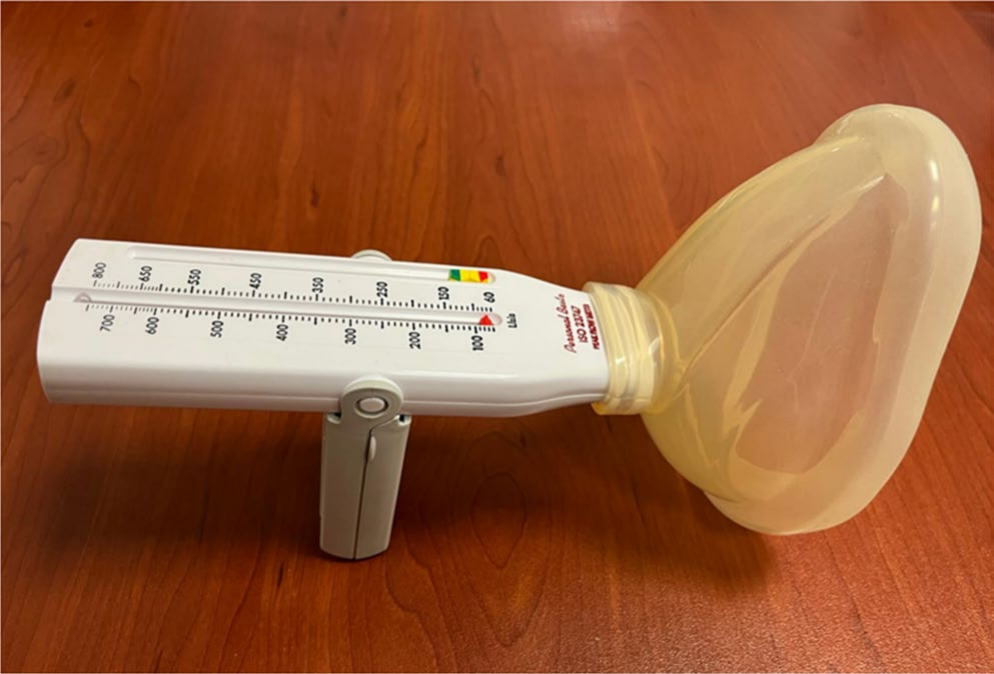
The analog peak flow meter used for PCF measurements

For the PCF procedure, participants were seated upright in a comfortable position, instructed to take a deep breath in, and then cough hard “as if something went down the wrong pipe” into a facemask connected to an analog peak flow meter (Philips Personal Best, ISO 23747; Fig. 1). This device was chosen because it is commercially available, low-cost, and comparable to those used in prior studies [15, 19–21, 27, 29, 30]. An assisting clinician securely positioned the facemask over the nose and mouth to maintain proper seal and measurement accuracy. Before the formal assessments, a live demonstration was performed for each participant. The procedure was performed five times, and the highest value obtained across the trials was recorded as PCF. The guidelines recommended by the European Respiratory Society were followed for the PCF measurements [35].

### Statistical analyses

The Statistical Package for the Social Sciences (SPSS Inc., Chicago, IL) software version 25 was used for all the analyses. The normality of the data was assessed using both visual methods (histograms and probability plots) and an analytical test (Kolmogorov–Smirnov). Demographic data and clinical characteristics of the participants were given descriptively. Since the data, including age, BMI, and PCF were normally distributed, an independent-samples t-test was performed to compare these data between the SG and CG. Since all tested FEES parameters in the SG were not normally distributed, the median (IQR) values were given for each IDDSI level related to the YPR-SRS and PAS scores. Receiver operator curves (ROC) analyses were applied to determine the optimal cutoff value for PCF. The optimal cutoff point for PCF was considered using the maximum of the Youden index (sensitivity + specificity – 1) and the maximized area under the curve (AUC) [36]. All the performed tests were two-tailed, and a p-value of < 0.05 was considered statistically significant.

## Results

The mean ± SD age was 66.64 ± 12.97 and 65.56 ± 16.39 in the SG and CG, respectively (*p* = 0.728). The mean ± SD BMI was 25.24 ± 4.63 in the SG and 26.32 ± 4.12 in the CG, with no significant differences between the groups (*p* = 0.272). Dysphagia etiology in the SG was heterogeneous, including stroke (*n* = 15, 33.3%), neurogenic (*n* = 10, 22.2%), head and neck cancer (*n* = 7, 15.6%), traumatic brain injury (*n* = 5, 11.1%), and other/unknown causes (*n* = 8, 17.8%). The general characteristics of the participants are outlined in Table 1.

**Table 1:** General characteristics of the participants

Variables	SG *N* = 45	CG *N* = 45	*p*-value
Age, years, (mean ± SD)	66.64 ± 12.97	65.56 ± 16.39	0.728
Gender, N (%)			
Female	20 (44.4)	20 (44.4)	1.000
Male	25 (55.6)	25 (55.6)	
BMI, kg/m^2^, (mean ± SD)	25.24 ± 4.63	26.32 ± 4.12	0.272
Diagnosis, N (%)			
Stroke	15 (33.3)		
Neurogenic	10 (22.2)		
Head and neck cancer	7 (15.6)	-	-
TBI	5 (11.1)		
Other/unknown	8 (17.8)		
PCF, L/min, (mean ± SD)	206.11 ± 74.22	287.78 ± 89.31	< 0.001

*SG* study group, *CG* control group, *BMI* body mass index, *TBI* traumatic brain injury, *PCF* peak cough flow

In the SG, the lowest median (IQR) YPR-SRS score of 1 (1–1) was observed for IDDSI level 7 in the pyriform sinus, while the higher median (IQR) YPR-SRS scores of 3 (2–4) were observed for both IDDSI levels 2 and 4 in the pyriform sinus. The lowest median (IQR) PAS score of 1 (1–1) was observed for IDDSI level 7, and the higher median (IQR) PAS score of 3 (1–8) was observed for IDDSI level 0. The detailed FEES characteristics of the SG were presented in Table 2.

**Table 2:** FEES characteristics of the SG

Parameters	SG (*N* = 45)	
	*N* (%)^a^	Median (IQR)
Swallowing efficiency (YPR-SRS)		
IDDSI 0-vallecula	33 (73.3)	2 (2–3)
IDDSI 0-pyriform sinus	33 (73.3)	3 (2–3)
IDDSI 2-vallecula	38 (84.4)	3 (2–3)
IDDSI 2-pyriform sinus	38 (84.4)	3 (2–4)
IDDSI 4-vallecula	44 (97.7)	3 (2–3)
IDDSI 4-pyriform sinus	44 (97.7)	3 (2–4)
IDDSI 7-vallecula	32 (71.1)	2 (1–3)
IDDSI 7-pyriform sinus	32 (71.1)	1 (1–1)
Swallowing safety (PAS)		
IDDSI 0	33 (73.3)	3 (1–8)
IDDSI 2	38 (84.4)	1 (1–5)
IDDSI 4	44 (97.7)	1 (1–5)
IDDSI 7	32 (71.1)	1 (1–1)

*SG* study group, *YPR*-*SRS* yale pharyngeal residue severity rating scale, *IDDSI* international dysphagia diet standardization initiative, *PAS* penetration-aspiration scale

^a^Valid percent of the swallowing trials

**Fig. 2:**
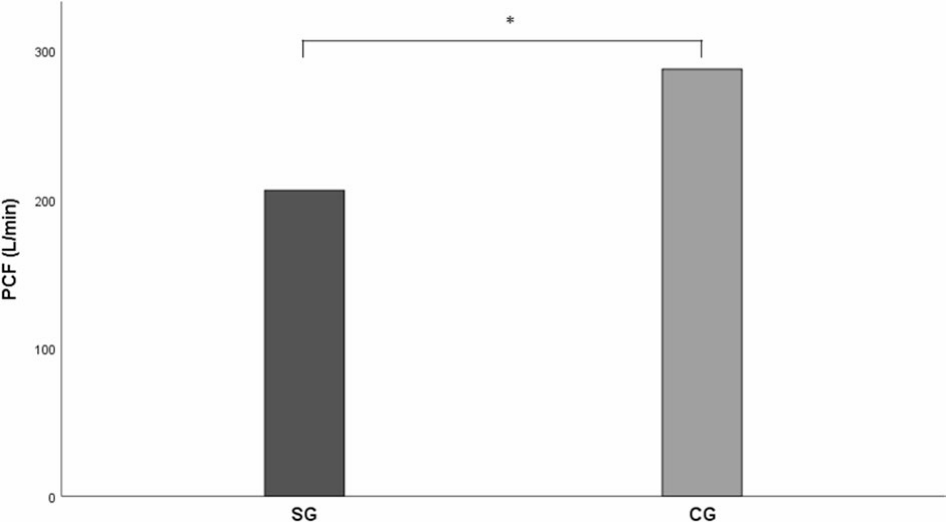
PCF comparison between the groups

**Fig. 3:**
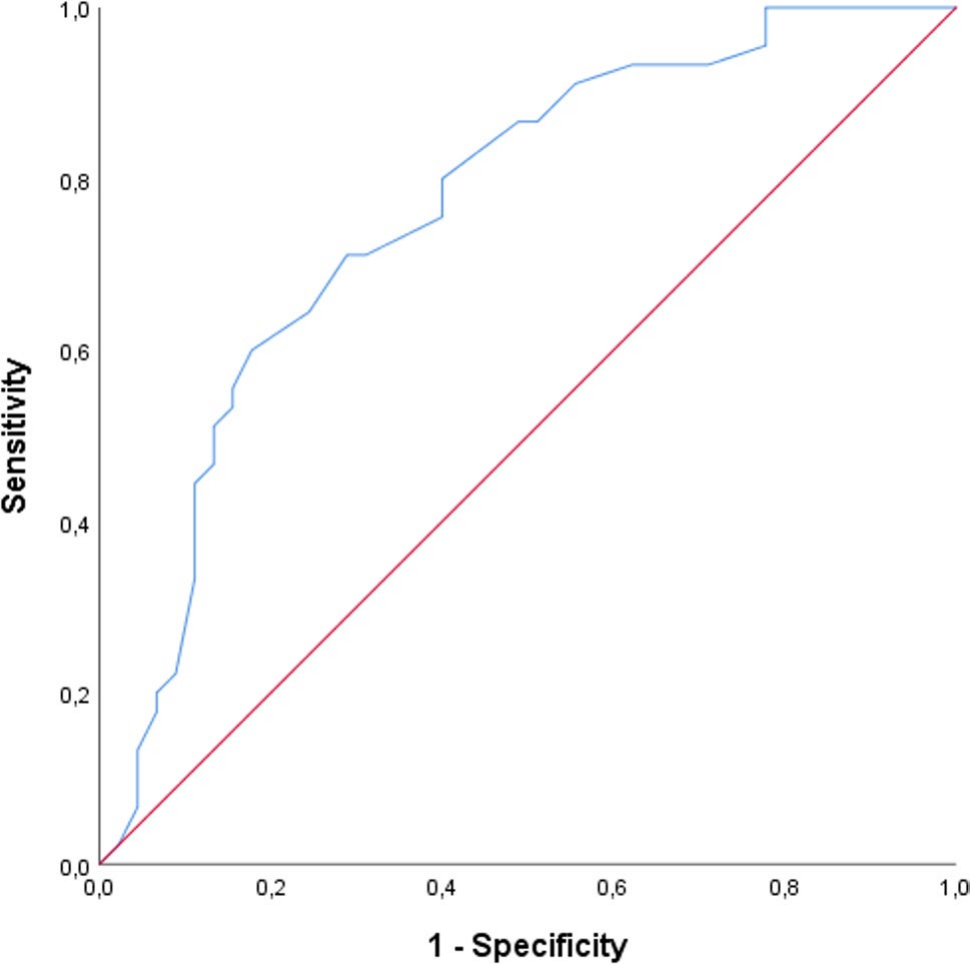
ROC analysis for PCF outcomes (SG vs. CG)

PCF values were 206.11 ± 74.22 and 287.78 ± 89.31 in the SG and CG, respectively. The PCF was significantly decreased in the SG than in the CG (*p* < 0.001), as illustrated in Fig. 2. ROC analysis revealed a cutoff PCF value of ≤ 252.5 L/min (AUC: 0.765, 95% CI: 0.666–0.865, 71.1% sensitivity and 71.1% specificity, *p* < 0.001) for predicting dysphagia (Table 3). Figure 3 illustrates the ROC graph for evaluating the discriminatory capacity of PCF for dysphagia.

**Table 3:** ROC analysis to determine the optimal cutoff value for PCF

Variables	AUC (95% CI)	Sig. *p*	Cutoff	Sens. %	Spec. %	NPV%	PPV%
SG vs. CG							
PCF	0.765 (0.666–0.865)	< 0.001	≤ 252.5	71.1	71.1	71.1	71.1

*SG* study group, *CG *control group, *AUC* area under the curve, *CI* confidence interval, *NPV* negative predictive value, *PPV* positive predictive value, *PCF* peak cough flow

## Discussion

This study investigated the relationship between cough and dysphagia in a heterogeneous patient population and determined a clinical cutoff value for PCF using a simple, portable peak flow meter. The findings demonstrated that individuals with dysphagia exhibited significantly lower PCF values than age- and sex-matched healthy controls, and a cutoff value below 252.5 L/min was identified to predict dysphagia with moderate sensitivity and specificity. These results support the close functional interplay between the swallowing and respiratory systems, highlighting the potential utility of PCF measurement as a simple adjunctive tool in clinical dysphagia evaluations.

The reduction in voluntary cough strength observed in this study is consistent with prior research conducted in more homogeneous patient groups. In stroke patients, Lee et al. [15] highlighted a close relationship between pharyngeal residue (swallowing efficiency) and voluntary PCF. In his studies, Hammond et al. [6, 16] reported that objective voluntary cough measurements can identify stroke patients with aspiration (swallowing safety) risk. Similarly, in studies conducted with patients with PD, Pitts et al. [5, 18] and Silverman et al. [20] demonstrated that voluntary cough parameters could reliably differentiate patients with evidence of penetration/aspiration from those without. Also, it was suggested that cough strength in the PD population is markedly impaired as disease severity worsens [20]. These findings were extended to individuals with ALS [4, 25], showing strong associations between voluntary cough measurements and swallowing safety outcomes [4]. Moreover, reduced cough function has also been reported in post-radiation head and neck cancer populations with aspiration [26, 27]. Our study builds on this body of evidence by including a heterogeneous cohort with diverse dysphagia etiologies, thereby reflecting real-world clinical dysphagia populations more accurately. However, findings from a recent study by Mir and Hegland [12] revealed that quantitative cough assessment tools such as peak flow meters were used by only 6.8% of clinicians, reflecting a limited integration of objective assessment techniques in clinical practice. Therefore, the present finding of reduced PCF in patients with diverse dysphagia etiologies underscores the potential value of quantitative cough measurements as a complementary component of comprehensive swallowing assessment in routine clinical practice.

It has been demonstrated that a minimum PCF of 160 L/min is required for effective airway clearance and successful extubation, whereas a value above 270 L/min is necessary to minimize pulmonary complications [37]. Based on this finding, Choi et al. [29] suggested that a PCF value of below 160 L/min (OR = 14.34; 95% CI, 1.84–111.60) is significantly associated with the development of pneumonia in patients with oropharyngeal dysphagia. One of the key findings of the present study is the identification of a PCF cutoff value of 252.5 L/min for predicting dysphagia. This threshold demonstrated moderate discriminatory accuracy (AUC = 0.765) with balanced sensitivity and specificity (71.1% each). As we know, only one prior study by Bianchi et al. [30] examined the PCF parameter in a heterogeneous patient population, and they reported that a PCF below 242 L/min was associated with increased pulmonary complications. However, relatively lower PCF thresholds were reported in other studies conducted with homogeneous patient populations. For example, a post-stroke PCF threshold of 151 L/min was stated for predicting dysphagia [17], while a cutoff value of 153 L/min was reported to be associated with aspiration in PD [22]. A higher PCF for the present heterogeneous patient population than for those homogeneous neurogenic populations was an expected finding. One plausible explanation relates to the reduced effectiveness of the expiratory phase of voluntary cough in neurogenic impairments, which may be due to the uncoordinated function of the respiratory muscles, laryngeal structures, and neural pathways. These consistent findings support the robustness of PCF measurement as a clinically meaningful parameter across patient populations.

Despite these promising findings, several considerations warrant discussion. First, although the inclusion of patients with diverse etiologies enhances the generalizability of the findings, subgroup analyses based on underlying pathology or swallowing efficiency and safety were not conducted due to limited sample size. Future research should explore whether optimal PCF thresholds differ by etiology, patients’ penetration/aspiration statuses, and residue amounts. Second, voluntary cough is an essential aspect of airway defense, but reflex cough—triggered by sensory stimulation—may provide complementary diagnostic information about the cough functions of patients with different dysphagia etiologies. Combining voluntary and reflex cough assessments may yield a more comprehensive evaluation of airway protection mechanisms in this population. Third, because individuals with known chronic respiratory diseases were excluded, the present findings cannot be directly extrapolated to populations in which low PCF primarily reflects underlying pulmonary dysfunction. In such cases, low PCF is more likely to indicate impaired airway clearance capacity that may coexist with, but does not necessarily cause dysphagia. Future studies should investigate the utility of PCF within cohorts that include patients with respiratory disease and examine whether PCF retains discriminant value for dysphagia after controlling for pulmonary function. Lastly, only vocal fold paralysis was examined in the current study, whereas other vocal fold mobility impairments and glottic configuration abnormalities were not systematically assessed or quantified as part of the study protocol. Thus, minor undetected differences in vocal fold mobility may have influenced the current PCF values. Therefore, incorporating detailed laryngeal imaging to explore the specific contribution of vocal fold mobility to PCF and its relationship with dysphagia-related outcomes would be valuable.

## Conclusions

The present study demonstrated that individuals with dysphagia exhibit significantly reduced voluntary peak cough flow compared with healthy controls, supporting the close functional relationship between the swallowing and respiratory systems. The identified cutoff value of 252.5 L/min may be useful for predicting dysphagia in patients with various etiologies. Given its simplicity, low cost, and portability, PCF may serve as a useful adjunctive tool for patients with dysphagia. The present findings suggest that incorporating PCF into routine clinical dysphagia assessments may facilitate early identification and intervention in dysphagia management, particularly in settings where instrumental assessments are not feasible.

## Data Availability

All data generated and/or analyzed during the current study are available from the corresponding author upon reasonable request.
